# Expression of Mucins in Different Entities of Salivary Gland Cancer: Highest Expression of Mucin-1 in Salivary Duct Carcinoma: Mucin-1 – highest expression in Salivary Duct Carcinoma

**DOI:** 10.1007/s12105-022-01448-3

**Published:** 2022-04-07

**Authors:** P. Wolber, M. Mayer, L. Nachtsheim, J. Prinz, J. P. Klußmann, A. Quaas, C. Arolt

**Affiliations:** 1 Department of Otorhinolaryngology, Head and Neck Surgery, Medical Faculty, University of Cologne, Cologne, Germany; 2 Institute of Pathology, Medical Faculty, University of Cologne, Cologne, Germany; 3 Department I of Internal Medicine, Center for Integrated Oncology Aachen Bonn Cologne Duesseldorf, University of Cologne, Cologne, Germany

**Keywords:** Salivary gland malignancy, Immunohistochemistry, Head and neck cancer, Targeted therapy, Mucin-1, Mucin-16, Mucin-5AC, Salivary duct carcinoma

## Abstract

Therapeutic options for advanced salivary gland cancer (SGC) are rare. Therefore, it was the aim of this study to investigate the extent and intensity of Mucin-1 (MUC1), Mucin-16 (MUC16), and Mucin-5AC (MUC5AC) as potential molecular targets using immunohistochemistry. The medical records of all patients who underwent primary surgery for salivary gland cancer with curative intent in a tertiary referral center between 1990 and 2018 were reviewed. Immunohistochemical staining for MUC1, MUC16, and MUC5AC was performed for all patients with sufficient formalin-fixed paraffin-embedded material, and a semi-quantitative combined score derived from the H-score for the cytoplasmatic, the membranous and the apical membrane was built for the most common entities of SGC. 107 patients with malignancies of the parotid (89.7%) and the submandibular gland (10.3%) were included. The most common entities were mucoepidermoid carcinoma (MuEp; n = 23), adenoid cystic carcinoma (AdCy; n = 22), and salivary duct carcinoma (SaDu; n = 21). The highest mean MUC1 combined score was found in SaDu with 223.6 (±91.7). The highest mean MUC16 combined score was found in MuEp with 177.0 (±110.0). The mean MUC5AC score was low across all entities. A higher MUC1 combined score was significantly associated with male gender (p = 0.03), lymph node metastasis (p < 0.01), lymphovascular invasion (p = 0.045), and extracapsular extension (p = 0.03). SaDu patients with MUC16 expression showed a significantly worse 5-year progression-free survival than those without MUC16 expression (p = 0.02). This is the first study to give a comprehensive overview of the expression of MUC1, MUC16, and MUC5AC in SGC. Since advanced SGCs lack therapeutic options in many cases, these results warrant in vitro research on therapeutic targets against MUC1 in SaDu cell lines and xenograft models.

## Introduction

Salivary gland carcinomas (SGC) are rare, representing 5–8% of all malignant tumors in the head and neck region [1, 2]. Most SGC originate from the parotid gland, followed by the submandibular gland and minor salivary glands [3]. Histopathological heterogeneity of SGC is high. Overall, more than 20 entities are described in the current WHO classification system [4]. Consequently, tumor biology and prognosis differ markedly when comparing the various entities. While the median 5-year-overall survival rate was described to be as high as 95% for low-grade mucoepidermoid carcinoma (MuEp) [5], salivary duct carcinoma (SaDu) has a median 5-year overall survival rate of lower than 45% [6].

Generally, the therapy of choice for SGC in absence of distant metastasis is surgical tumor resection. Furthermore, an ipsilateral neck dissection is recommended in advanced-stage carcinomas (T3–4), in high-grade carcinomas, and in carcinomas with clinical, sonographic, or radiological suspicion of loco-regional nodal involvement [7, 8]. In adenoid cystic carcinoma (ACC), advanced-stage tumors, high-grade tumors, positive/close margins or in case of lymph node metastasis, (lympho-)vascular, and perineural invasion, adjuvant radiation therapy is indicated [8]. In case of unresectable or metastatic SGC, mainly platinum-based palliative chemotherapy regimens, i.e., the SGC regimen consisting of cyclophosphamide, doxorubicin, and cisplatin, are applied [9]. However, response rates and survival are rather low, and toxicity can be extensive [10]. Therefore, an increasing number of molecular targets for a targeted therapy approach in advanced SGC has been identified over the recent years. Molecular targets with promising clinical data are HER-2, tyrosine kinase, EGFR, c-kit, the NTRK fusion protein, and the androgen receptor [10, 11]. However, in clinical practice tumors are still often lacking a molecular target leading to limited therapeutic options in these cases. Hence, to provide more patients with an option for a tailored therapy, the identification of further molecular targets in SGC is of utmost importance. Molecular targets are of particular importance among entities with a high rate of distant metastasis such as SaDu and adenoid cystic carcinoma (ACC) which are reported to metastasize in 52–82% and 24% of cases, respectively [12–14].

Mucins are a family of glycosylated proteins produced by epithelial tissues in humans. Mucin-1 (MUC1), also known as CA 15–3, is a type I transmembrane glycoprotein with a cytoplasmatic tail serving as an adaptor protein connected with kinases and further cell signaling proteins leading to cell proliferation, infiltration into the extracellular matrix, deregulation of apoptosis and changes in the adhesion state of the cell [15, 16]. It has been shown that in cancer cells with MUC1-overexpression phosphatidylinositol 3-kinase (PI3K), mitogen-activated protein kinase (MAPK) and wingless type (Wnt) pathways are overstimulated [17]. AS1402, a humanized immunoglobin monoclonal antibody binding to the MUC1-N tandem, showed antibody-dependent cytotoxicity against MUC-1-positive breast cancer cells in a phase I trial [18]. Moreover, anti-MUC1 vaccines have shown promising results in phase I/II trials but have failed to show survival benefits compared to standard therapy in phase III trials such as Tecemotide in patients with stage III non-small-cell lung cancer or PANVAC C/F in patients with stage IV pancreatic cancer [19, 20]. Therefore, MUC-1 represents an interesting molecular target for the treatment of SGC.

Mucin-16 (MUC16), also known as CA-125, is another type 1 transmembrane glycoprotein, routinely used as a tumor marker in ovarian cancer and found in the epithelia of several organs such as the trachea, the ocular surface, the abdominal cavity, and the female reproductive tract [21]. MUC16 was shown to be associated with growth and metastasis [21] of cancer cells through inhibition of the function of natural killer cells [22], the interaction with the janus kinase 2 (JAK2) leading to upregulation of the expression of the stem cell genes [23], and other molecular mechanisms. The anti-MUC16 antibody Oregovomab compared to placebo showed no survival benefit for patients with recurrent ovarian cancer after first-line therapy in the whole study group but a significantly greater disease-free survival in a subpopulation more amenable to immunotherapy in a randomized, double-blind study [24].

In opposite to type 1 transmembrane MUC1 and MUC16, Mucin-5AC (MUC5AC) is a type 2 secreted mucin mainly found in the mucus of the respiratory tract. A significant association between MUC5AC and a worsened survival in adenocarcinoma of the lung has been reported in multiple studies [25, 26]. In the animal model tumorigenesis was significantly reduced in mice with lacking MUC5AC compared to controls which identifies MUC5AC as a potential molecular target [27].

The aim of the current study was to investigate the expression of the potential molecular targets MUC1, MUC16, and MUC5AC in different entities of SGCs using a combined score of apical, membranous, and cytoplasmatic expression in immunohistochemistry.

## Methods

### Cohort

All patients with primary salivary gland carcinoma of the parotid or submandibular gland who had undergone primary surgery with curative intent at the Department of Otorhinolaryngology, Head and Neck Surgery, University of Cologne between 1990 and 2018 with sufficient formalin-fixed paraffin-embedded (FFPE) material of the primary tumors were included in the analysis. The study was performed according to the regulations of the Ethics Committee of the University of Cologne.

Demographic characteristics and histopathological data were retrieved from clinical records and histopathological reports with respect to tumor characteristics including the stage of disease at the time of diagnosis according to the AJCC TNM staging system (8th edition, 2020) [28]. Four tissue cylinders per case were used to build tissue microarrays (TMA) as described before [29]. All histologic diagnoses were reviewed as described before [30]. Briefly, we used a selection from the following tests to resolve unequivocal diagnoses and to minimize the number of adenocarcinomas NOS in the cohort: immunohistochemical stainings for CK7, p63, NOR-1, SOX10, androgen receptor and HER2, FISH break-apart probes targeting *MYB, MYBL1, PRKD1, PRKD2, PRKD3, EWSR1, MAML2* and *ETV6* genes as well as Sanger sequencing of *PRKD1* hotspot mutations [31].

### Immunohistochemistry and Assessment of Mucin Expression

Briefly, tissue slides were stained with antibodies against MUC1 (clone EP85, rabbit, 1:500 pretreatment with EDTA buffer, Epitomics, Burlingame, CA, USA), MUC16 (CA12.5, clone M11, mouse, 1:200 pretreatment with citrate buffer, Dako/Agilent, Santa Clara, CA, USA), and MUC5AC (clone MUC5AC/917, mouse, 1:500, pretreatment with EDTA buffer, Abcam, Cambridge, UK). All IHC stainings were carried out with a Leica BOND-MAX stainer (Leica Biosystems, Wetzlar, Germany) in accordance with the manufacturer’s protocol. Counterstaining was done using haematoxylin and bluing reagent.

First, all TMA sections were screened to explore different staining patterns. Cytoplasmatic, membranous and apical (luminal membrane stained, max. 2 sides of the cell membrane) staining patterns were observed. Then, two pathologists with special expertise in the field of SGC (CA, AQ) assesed the expression of each pattern using the Histo-score (H-score) [32], which was calculated as follows: First, three different levels of staining intensity (strong, moderate, weak) were defined. Then, the percentage of cells stained at each intensity level was multiplied with 1 in case of weak staining, with 2 and 3 in case of moderate and strong staining, respectively. Consecutively, the H-Score ranges between 0 (0% cells stained) and 300 (100% * 3). The consensus (by CA and AQ) overall percentage in the four tissue cores was used for H-score calculation. A combined score was calculated as the sum of the three H-scores (cytoplasmatic, membranous, apical; theoretical range 0–900).

### Statistical Analysis

Statistical analyses were performed using SPSS software version 28.0 (IBM, Armonk, NY). Distribution was tested using the Shapiro–Wilk-Test. Spearman’s rank correlation coefficient was used to measure the strength of correlation between non-normally distributed variables. The Mann–Whitney–U-test was used to compare differences between independent groups for metric, non-normally distributed variables. Kaplan–Meier method with 95% confidence intervals was used to test for progression-free survival (PFS) probability rates. For this, statistical significance was tested by using the log-rank test. PFS was defined as the time interval between the end of treatment and the date of progression of the specific disease. A p-value < 0.05 was considered statistically significant. R studio (version 2021.09.1) was used for visualization of box plots (ggplot2 package).

## Results

Overall, 107 patients with primary cancer of the parotid- (n = 96; 89.7%) and submandibular gland (n = 11; 10.3%) were included (Table 1). The mean age was 54.9 (± 17.9) years. Fifty-six patients (52.3%) were female. Fifty-three (49.5%) patients had a pT1-2 tumor, whereas 52 (48.6%) patients showed a pT3-4 tumor. Most patients (n = 71; 66.4%) showed tumor-free cervical lymph nodes (pN0).

**Table 1:** Localization of the primary, demographic data, histopathological data, and mean MUC1, MUC16, MUC5AC combined scores for the whole cohort and for the most frequent entities

	All n = 107	MuEp n = 23	AdCy n = 22	SaDu n = 21	ACC n = 10	ANOS n = 9	EpMy n = 7	SecC n = 7	OTH n = 8
Localization									
Parotid gland	96 (89.7)	23 (100.0)	16 (72.7)	18 (85.7)	10 (100.0)	9 (100.0)	7 (100.0)	6 (85.7)	7 (87.5)
Submandibular gland	11 (10.3)	0 (0.0)	6 (27.3)	3 (14.3)	0 (0.0)	0 (0.0)	0 (0.0)	1(14.3)	1 (12.5)
Demographics									
Male	51 (47.7)	6 (26.1)	9 (40.1)	15 (71.4)	5 (50.0)	4 (44.4)	5 (50.0)	4 (57.1)	3 (37.5)
Female	56 (52.3)	17 (73.9)	13 (59.9)	6 (28.6)	5 (50.0)	5 (55.6)	5 (50.0)	3 (42.99)	5 (62.5)
Age	54.9 ± 17.9	42.3 ± 17.3	51.5 ± 14.3	66.0 ± 11.9	51.4 ± 18.6	63.7 ± 15.4	61.0 ± 18.8	48.6 ± 20.0	66.1 ± 16.4
Histopathological parameters T-stage									
T1-2	53 (49.5)	14 (60.1)	10 (45.5)	7 (33.3)	5 (50.0)	6 (66.7)	4 (57.1)	5 (71.4)	2 (25.0)
T3-4	52 (48.6)	8 (34.8)	11 (52.4)	14 (66.7)	5 (50.0)	3 (33.3)	3 (42.9)	2 (28.6)	6 (75.0)
N/A	2 (1.9)	1 (5.1)	1 (2.1)	0 (0.0)	0 (0.0)	0 (0.0)	0 (0.0)	0 (0.0)	0 (0.0)
N-stage									
N0	71 (66.4)	19 (82.6)	14 (63.6)	3 (14.3)	8 (80.0)	6 (66.7)	7 (100.0)	6 (85.7)	8 (100.0)
N+	33 (30.8)	3 (13.0)	7 (31.2)	18 (85.7)	1 (10.0)	3 (33.3)	0 (0.0)	1 (14.3)	0 (0.0)
N/A	3 (2.8)	1 (4.3)	1 (5.2)	0 (0.0)	1 (10.0)	0 (0.0)	0 (0.0)	0 (0.0)	0 (0.0)
Vascular invasion									
V0	89 (83.2)	20 (87.0)	16 (72.3)	19 (90.5)	9 (90.0)	6 (66.7)	7 (100.0)	6 (85.7)	6 (75.0)
V1	8 (7.5)	2 (8.7)	0 (0.0)	1 (4.7)	0 (0.0)	3 (33.3)	0 (0.0)	0 (0.0)	2 (25.0)
N/A	10 (9.3)	1 (4.3)	6 (27.3)	0 (0.0)	1 (10.0)	0 (0.0)	0 (0.0)	0 (0.0)	0 (0.0)
Perineural invasion									
Pn0	61 (57.0)	18 (78.2)	12 (54.5)	10 (47.6)	7 (70.0)	5 (55.6)	7 (100.0)	7 (100.0)	5 (62.5)
Pn1	36 (33.6)	5 (21.3)	6 (27.3)	10 (47.6)	3 (30.0)	4 (44.4)	0(0.0)	0 (0.0)	2 (25.0)
N/A	10 (9.4)	0 (0.0)	3 (13.6)	1 (4.8)	0 (0.0)	0 (0.0)	0 (0.0)	0 (0.0)	1 (12.5)
Lymphovascular invasion									
L0	85 (79.4)	21 (91.3)	16 (72.3)	11 (52.4)	8 (80.0)	8(88.9)	7 (100.0)	7 (100.0)	7 (87.5)
L1	13 (12.1)	1 (4.3)	0 (0.0)	9 (42.9)	1 (10.0)	1 (11.1)	0 (0.0)	0 (0.0)	1 (12.5)
N/A	9 (8.5)	1(4.3)	6 (27.3)	1 (4.7)	1 (10.0)	0 (0.0)	0 (0.0)	0 (0.0)	0 (0.0)
Extracapsular extension									
ECE−	82 (76.6)	21 (91.3)	13 (59.1)	10 (47.6)	8 (80.0)	8 (88.9)	7 (100.0)	7 (100.0)	8 (100.0)
ECE+	16 (15.0)	1 (4.3)	3 (13.6)	10 (47.6)	1 (10.0)	1 (11.1)	0 (0.0)	0 (0.0)	0 (0.0)
N/A	9 (8.4)	1 (4.3)	6 (27.3)	1 (4.8)	1 (10.0)	0 (0.0)	0 (0.0)	0 (0.0)	0 (0.0)
MUC 1 comb. score	109.1 ± 107.4	146.3 ± 80.1	26.9 ± 45.1	223.6 ± 91.7	29.1 ± 60.9	136.7 ± 106.3	73.6 ± 78.2	140.7 ± 110.4	0.0 ± 0.0
MUC 16 comb. score	74.6 ± 104.4	177.0 ± 101.0	33.6 ± 44.7	63.0 ± 112.5	81.1 ± 110.9	85.3 ± 134.0	27.4 ± 37.7	8.4 ± 9.6	1.6 ± 3.5
MUC 5AC comb. score	5.3 ± 23.3	24.0 ± 46.2	0.0 ± 0.0	0.6 ± 2.6	0.0 ± 0.0	0.6 ± 1.7	0.0 ± 0.0	0.0 ± 0.0	0.0 ± 0.0

*n* number of patients, *()* percentages, ± standard deviation, *MuEp* mucoepidermoid carcinoma, *AdCy* adenoid cystic carcinoma, *SaDu* salivary duct carcinoma, *ACC* acinic cell carcinoma, *ANOS* adenocarcinoma not otherwise specified, *EpMy* epithelial-myoepithelial carcinoma, *SeC* Secretory carcinoma, *OTH* Others

The most common entity was mucoepidermoid carcinoma (MuEp; n = 23; 21.5%), followed by adenoid cystic carcinoma (AdCy; n = 22; 20.6%), salivary duct carcinoma (SaDu; n = 21; 19.6%), acinic cell carcinoma (ACC; n = 10; 9.3%), adenocarcinoma NOS (ANOS; n = 9; 8.4%), epithelial-myoepithelial carcinoma (EpMy; n = 7; 6.5%), and secretory carcinoma (SecC; n = 7; 6.5%). Other rare entities (OTH; n = 8; 7.5%) were basal cell carcinoma (n = 3; 2.8%), myoepithelial carcinoma (n = 2; 1.9%), oncocytic carcinoma (n = 1; 0.9%), carcinoma ex pleomorphic carcinoma (n = 1; 0.9%), carcinosarcoma (n = 1; 0.9%), and polymorphous adenocarcinoma (n = 1; 0.9%). Additional histopathological data is presented in Table 1.

A significantly positive correlation was found between cytoplasmatic and membranous staining in MUC1, MUC 16, and MUC5AC (p-values < 0.01, < 0.01, < 0.01; Spearman’s ρ = 0.43, = 0.67, = 0.83, respectively). Further, a significantly positive correlation was found between cytoplasmatic and apical staining in MUC1 and MUC 16 (p-values < 0.01, < 0.01; Spearman’s ρ = 0.55, = 0.48, respectively). A significantly positive correlation was found between apical and membranous staining in MUC16 (p = 0.03; Spearman’s ρ = 0.21). No significant correlation was found between apical and membranous staining in MUC1 (p = 0.69; Spearman’s ρ = − 0.04). Due to the low apical expression of MUC5AC no analyses were performed for the correlation between apical and cytoplasmatic or apical and membranous staining in MUC5AC. Since most of the correlations between the apical, membranous, and cytoplasmatic H-scores were significant, the mean combined scores are presented in the following to display a comprehensive expression pattern of the whole cell.

**Fig. 1:**
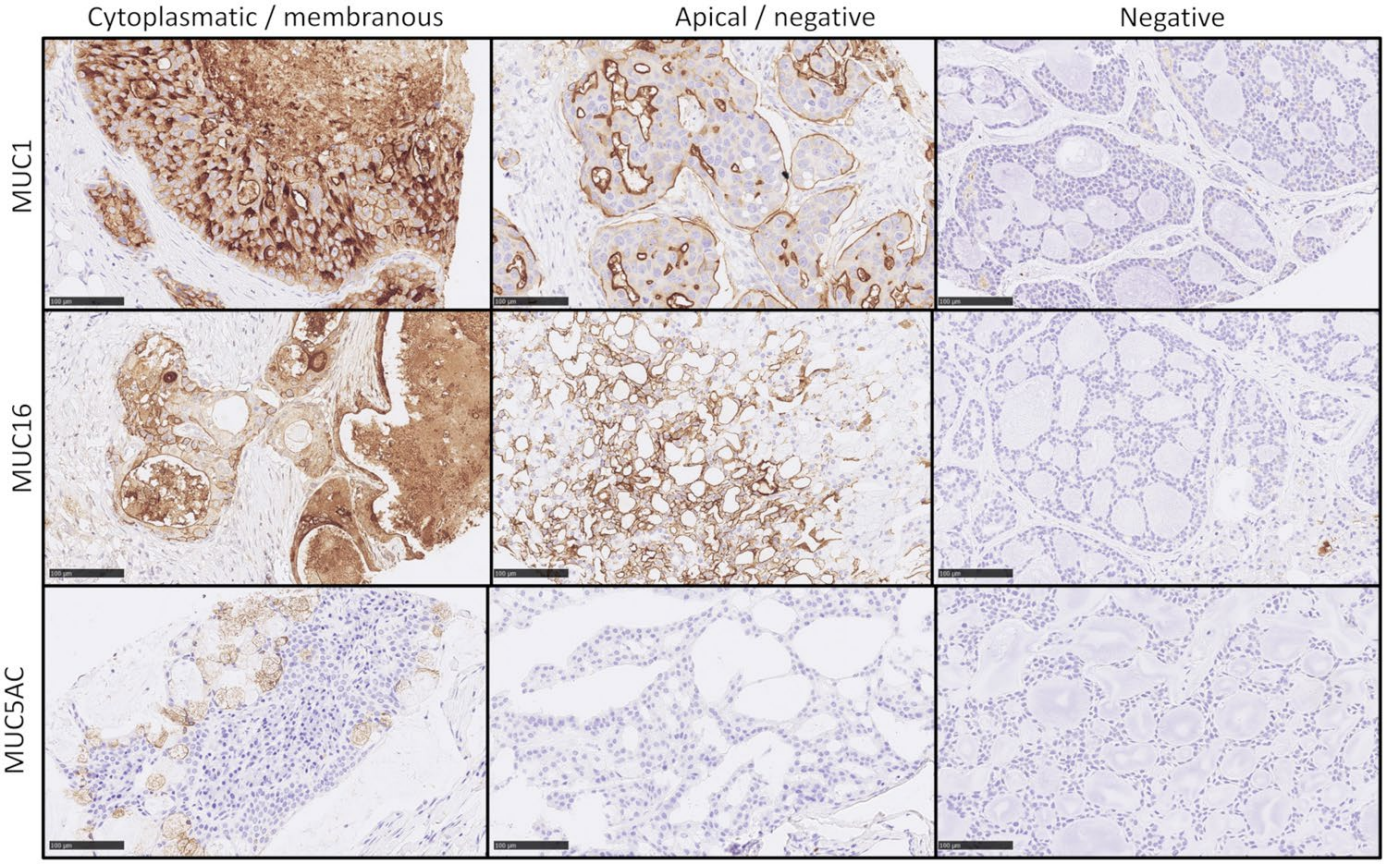
Protein expression of MUC1 (top row) MUC16 (middle row) and MUC5AC (bottom row). From left to right; Top row: Weak to strong mixed cytoplasmatic and membranous expression of MUC1 in a SaDu with classical comedonecrosis-like growth pattern, a moderate to strong apical staining in another SaDu case and a very weak staining in AdCy. Middle row: Cytoplasmatic and membranous MUC16 expression in a MuEp, strong apical staining pattern in an Acin case and negativity for MUC16 in another AdCy. Bottom row: MUC5AC staining was nearly exclusive to goblet cells in MuEp, other carcinomas such as Sec (center) and AdCy (right) were MUC5AC negative

The protein expression of MUC1, MUC16, and MUC5AC is showed for exemplary cases in Fig. 1.

**Fig. 2:**
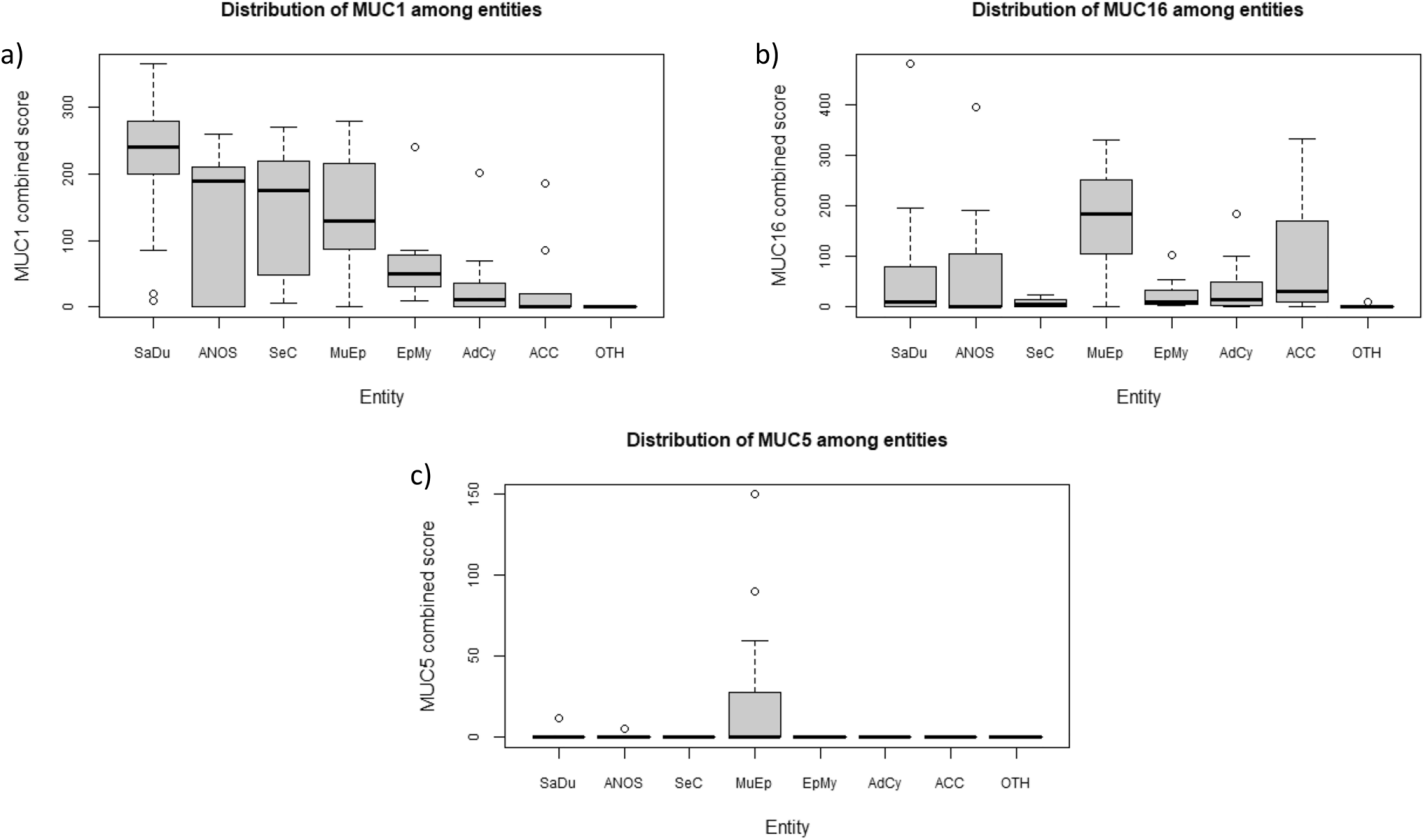
Box plots displaying the distribution of **a** the MUC1 combined score, **b** the MUC16 combined score, and **c** the MUC 5AC combined score among the most frequent entities. *SaDu* Salivary duct carcinoma, *SeC* Secretory carcinoma, *ANOS* adenocarcinoma not otherwise specified, *MuEp* Mucoepidermoid carcinoma, *EpMy* Epithelial-myoepithelial carcinoma, *AdCy* adenoid cystic carcinoma, *ACC* Acinic cell carcinoma, *OTH* Others

The distribution of the combined scores for MUC1, MUC16, and MUC5AC is displayed in Fig. 2.

The mean combined score for MUC1 among all included entities was 109.1 (± 107.4). The highest mean MUC1 combined score was found in SaDu being as high as 223.6 (± 91.7), followed by MuEp (146 ± 80.1), SecC (140.7 ± 110.4), ANOS (136.7 ± 106.3), EpMy (73.6 ± 78.2), ACC (29.1 ± 60.9), and AdCy (26.9 ± 45.1). In total, 81 SGC (75.7%) showed a MUC1 expression.

The mean combined score for MUC16 among all included entities was 74.6 (± 104.4). The highest mean MUC16 combined score was found in MuEp with a score of 177.0 (± 101.0), followed by ANOS (85.3 ± 134.0), ACC (81.1 ± 110.9), SaDu (63.0 ± 112.5), AdCy (33.6 ± 44.7), EpMy (27.4 ± 37.7), and SeC (8.4 ± 9.6). In total, 80 SGC (74.8%) showed a MUC16 expression.

The mean combined score for MUC5AC among all included entities was 5.3 (± 23.3). The mean combined score for MUC5AC was generally low compared to the MUC1 and MUC16 combined scores. MUC5AC was detected in MuEp with a mean combined score of 24.0 (± 46.2), in SaDu with a very low mean score of 0.6 (± 2.6), and in ANOS with 0.6 (± 1.7). MUC5AC was not found in MuEp, ACC EpMy, and SeC. In total, 10 SGC (9.3%) showed a MUC5AC expression.

Table 2 illustrates the association between the mean combined MUC1, MUC16, and MUC5AC scores and basic demographic as well as histopathological data. A higher MUC1 combined score was statistically significantly associated with male gender (p = 0.03), pathological N+ stage (p < 0.01), lymphovascular invasion (p = 0.045), and extracapsular nodal extension (p = 0.03). No statistically significant association was found between the MUC1 combined score and tumor localization, T-stage, vascular invasion and perineural invasion. Female gender was significantly associated with a higher MUC16 combined score (p = 0.03) and a higher MUC5AC combined score (p = 0.01), respectively. There was no further statistically significant association between the MUC16 or MUC5AC combined score and the examined variables. The statistical association between the mean MUC combined scores of the most frequent entities and localization/demographic/histopathological data was not investigated due to insufficient sizes of the subgroups.

**Table 2:** Statistical association between localization of the primary tumor, sex, histopathological data and the mean MUC1, MUC16 and MUC5AC scores

	MUC1 comb. score		MUC16 comb. score		MUC5AC comb. score	
Parotid gland	111.87 (± 11.11)	p = 0.33	75.66 (± 10.21)	p = 0.26	5.94 (± 2.50)	p = 0.57
Submandibular gland	84.82 (± 28.73)	U = 433.00	64.82 (± 42.82)	U = 472.50	0.00 (± 0.00)	U = 473.00
Male	134.12 (± 16.83)	**p = 0.03**	43.09 (± 9.90)	**p = 0.03**	0.99 (± 0.99)	**p = 0.01**
Female	86.25 (± 11.94)	U = 1090.50	103.12 (± 16.21)	U = 1776.00	10.10 (± 4.21)	U = 1634.00
T1/2	131.99 (± 15.2)	p = 0.12	95.31 (± 15.99)	p = 0.16	10.27 (± 4.76)	p = 0.18
T3/4	104.57 (± 17.41)	U = 1135.50	52.72 (± 12.11)	U = 1158.50	1.52 (± 1.00)	U = 1271.50
N0	91.31 (± 11.77)	**p < 0.01**	79.63 (± 12.10)	p = 0.31	7.86 (± 3.35)	p = 0.12
N +	156.65 (± 19.61)	U = 1564.50	64.88 (± 19.46)	U = 1025.00	0.36 (± 0.36)	U = 1056.00
V0	123.30 (± 11.97)	p = 0.19	74.27 (± 10.47)	p = 0.59	6.63 (± 2.78)	p = 0.32
V1	66.43 (± 34.56)	U = 256.00	86.00 (± 53.09)	U = 397.00	0.00 (± 0.00)	U = 316.00
Pn0	110.23 (± 13.21)	p = 0.36	89.11 (± 13.92)	p = 0.14	8.78 (± 3.97)	p = 0.23
Pn1	134.18 (± 21.30)	U = 1221.00	50.94 (± 14.17)	U = 902.00	1.53 (± 1.22)	U = 1012.50
L0	110.04 (± 11.58)	**p = 0.045**	81.89 (± 11.67)	p = 0.21	6.98 (± 2.99)	p = 0.71
L1	174.23 (± 37.88)	U = 742.50	33.69 (± 14.56)	U = 902.00	0.92 (± 0.92)	U = 534.00
ECE−	109.58 (± 12.01)	**p = 0.03**	81.40 (± 11.34)	p = 0.14	7.40 (± 3.10)	p = 0.09
ECE+	164.44 (± 30.53)	U = 881.00	45.13 (± 25.10)	U = 483.00	0.00 (± 0.00)	U = 576.00

± Standard deviation, Significance level p < 0.05, Statistically significant results marked in bold, U = Mann–Whitney–U

### Survival

**Fig. 3:**
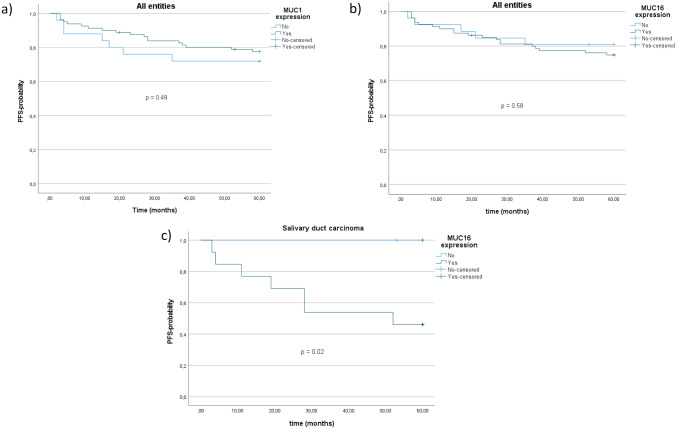
Kaplan–Meier curves and p-values of log-rank tests for **a** MUC1 expression among all entities, **b** MUC16 expression among all entities, **c** MUC16 expression among patients with salivary duct carcinoma. *PFS* Progression-free survival. All entities = Salivary gland cancer cohort (n = 107)

After a mean follow-up of 50.1 months (± 18.9) the 5-year progression-free survival (PFS) among all patients was 76.4%. PFS did not differ significantly between patients with and without MUC1 expression among all entities (p = 0.49; Fig. 3a). No significant PFS differences were found regarding MUC1 expression in the most frequent subgroups MuEp (p = 0.89), AdCy (p = 0.65), and SaDu (p = 0.11). Moreover, PFS did not differ significantly between patients with and without MUC16 expression among all entities (p = 0.58; Fig. 3b). Interestingly, SaDu patients with MUC16 expression (46.2%) had a significantly decreased 5-year-PFS compared to those without MUC16 expression (100.0%; p = 0.02; Fig. 3c). No significant PFS differences were found between patients with and without MUC16 expression in MuEp (p = 0.52) and AdCy (p = 0.77). No survival analyses were performed for MUC5AC due to the generally low expression.

## Discussion

The current study aimed to determine the expression rate of Mucin-1 (MUC1), Mucin-16 (MUC16), and Mucin-5AC (MUC5AC) in salivary gland carcinomas (SGC) using immunohistochemistry. To the best of our knowledge, this is the first study to display the expression of MUC1, MUC16, and MUC5AC among various entities of SGC using a semi-quantitative combined score derived from the H-score.

The most frequent entities in this series were mucoepidermoid carcinoma (MuEp) (n = 23; 21.5%), followed by adenoid cystic carcinoma (AdCy) (n = 22; 20.6%), salivary duct carcinoma (SaDu) (n = 21; 19.6%), and acinic cell carcinoma (ACC) (n = 10; 9.3%). This is partly in line with the histopathological distribution of SGC found in population-based studies showing MuEp, AdCy, and ACC to be the most frequent entities [33], whereas SaDu is found in up to 6% of SGC [34].

The highest mean MUC1 combined score among all included entities was found in SaDu being as high as 223.6 (± 91.7), followed by MuEp (146.3 ± 80.1), and SecC (140.7 ± 110.4). This is of particular interest as SaDu shows distant metastases in 52–82% of cases [12, 13] with a particularly poor median 5-year overall survival rate of lower than 45% [6]. Therefore, MUC1 seems to qualify as a therapeutic target in recurrent or metastatic SaDu. As mentioned above, various therapeutic agents targeting MUC1, such as monoclonal antibodies (MAb), small molecule inhibitors, and vaccines, have already been developed. The MAb AS1402, that binds to MUC1-N (the extra-cellular domain of the MUC1-complex) showed cellular toxicity in MUC1 positive breast cancer cells but failed to show efficacy in combination with letrazole compared to letrazole alone in a Phase III study among patients with metastatic breast cancer [18]. A potential reason for the missing efficacy is that the MUC1-N subunit is loosely connected to the MUC1-C subunit, the membrane-bound domain of MUC1. After shedding from the cell surface MUC1-N forms a thick layer on the outside of the tumor cell and is no longer connected to cytoplasmatic signal transduction [35]. However, the effect of MAb binding to MUC1-N has not been investigated in SGC, to date. In more recent studies, the cytoplasmatic region of the MUC1-C subunit has been targeted by small molecule inhibitors, which are oligonucleotides derived from RNA/DNA with specific amino acid sequences matching the phosphorylation sites in MUC1-C [35]. One of these small molecule inhibitors targeting the MUC1-C subunit is GO-201. Treatment of MUC1-positive prostate cancer cells with GO-201 lead to reduced cell proliferation and necrotic cell death, whereas no such effect was observed in MUC1-negative prostate cancer cells. Moreover, GO-201 lead to prolonged lack of recurrence and complete tumor regression in the mouse model [36]. Similarly, GO-201 resulted in necrosis, loss of tumorigenicity, and prolonged regression of tumor growth in MUC1-positive breast cancer cells in vitro as well as in the mice model [37]. GO-201 has not been studied for MUC1-positive SGC, yet. Besides passive immunotherapy, there are extensive approaches targeting MUC1 by active immunotherapy, i.e., vaccines. Whereas phase III trials with the anti-MUC1 vaccines Tecemotide in patients with stage III non-small-cell lung cancer and PANVAC C/F in patients with stage IV pancreatic cancer did not show survival benefits compared to standard therapy [19, 20], various studies are investigating anti-MUC1 peptide, carbohydrate, DNA, and dendritic cells (DC) vaccines [35]. For example, the peptide vaccine oxidized-mannan-MUC1 showed a significantly reduced recurrence rate in the verum versus the placebo group (12.5% vs. 60.0%) among patients with stage II breast cancer in a pilot phase III study with a long-term follow-up of up to 15 years [38]. Moreover, an anti-MUC1 DC vaccine showed a significantly prolonged survival among patients with immunohistochemically MUC1-positive advanced or metastatic breast or lung cancer compared to MUC1-negative patients [39]. date, neither MAb, nor small molecule inhibitors or vaccines against MUC1 have been studied among patients with SGC.

The present data shows that in our cohort male gender (p = 0.03), the presence of lymph node metastasis (p < 0.01), lymphovascular invasion (p = 0.045), and extracapsular extension (p = 0.03) were significantly associated with a higher mean MUC1 combined score among all SGC. The results are in line with a study from Liu et al. who showed a statistically significant association between a high expression of MUC1 and male gender as well as lymph node metastasis in a series of MuEp [40] as well as with data from Alos et al. who likewise showed an association between a high MUC1 expression and lymph node metastasis in MuEp [41]. Also, MUC1 serves as an adaptor protein leading to cell proliferation, infiltration into the extracellular matrix, and deregulation of apoptosis [15, 16]. These findings suggest that MUC1 expressing SGC might represent a subset of tumors with a higher likelihood of lymphovasular invasion, lymph node metastasis, extracapsular extension and consequently, a particular need for targeted therapy. However, it must be mentioned that the subgroup of SaDu, which showed the highest mean combined MUC1 score among all entities, consisted of 71.4% male patients, 85.7% of patients with lymph node metastasis, 42.9% of patients with lymphovascular invasion, and 47.6% of patients with extracapsular extension. Thus, the association between these variables and a higher mean MUC1 combined score in our cohort might be caused by the distribution if these variables in the subgroup of SaDu. The statistical association between the mean MUC combined scores and demographic/histopathological data within the subgroups was not investigated due to insufficient sizes of the subgroups.

The highest mean MUC16 combined score was found in MuEp with 177.0 (± 101.0), followed by ANOS (85.3 ± 134.0), ACC (81.1 ± 110.9), SaDu (63.0 ± 112.5), and AdCy (33.6 ± 44.7). MUC16 is mainly known as a routinely used tumor marker CA-125 in ovarian cancer. However, it was also shown to be associated with growth and metastasis [21] of cancer cells through inhibition of the function of natural killer cells [22] and the interaction with the janus kinase 2 (JAK2). Therefore, it potentially qualifies as a molecular target. The MAb Oregovumab and vaccines targeting MUC16 have been investigated among patients with ovarian cancer. In a phase II study Oregovomab compared to placebo showed no survival benefit for patients with recurrent ovarian cancer after first-line therapy in the whole study group but a significantly greater disease-free survival in a subgroup with microscopic or small residual disease after primary surgical debulking, favorable response to chemotherapy, and normalized but measurable CA-125 [24]. Another recently published phase II study comparing chemotherapy versus chemotherapy plus Oregovumab among stage III/IV ovarian cancer patients proved a significantly increased progression free survival among the chemotherapy plus Oregovumab group [42]. To date, no study has evaluated the use of Oregovumab among patients with SGC. Oregovumab may be favorably studied among patients with recurrent or metastatic MuEp as this entity showed the highest mean MUC16 combined score in the present study. However, MuEp mostly present as slowly progressive tumors with an excellent 10-year overall survival of 86.6% which can be treated surgically [43]. Therefore, advanced-stage MuEp are rare.

A higher mean MUC16 combined score was statistically significantly associated with the female gender. This is most likely due to the finding that MuEp showed the highest mean MUC16 combined score and most patients with MuEp in this series (73.9%) were female. No statistically significant association between a higher MUC16 combined score and histopathological data or localization was found.

The mean MUC5AC combined score was generally low compared to the MUC1 and MUC16 combined scores. MUC5AC was detected in MuEp with a mean combined score of 24.0 (± 9.6) and in SaDu with a very low mean score of 0.6 (± 0.6), and in ANOS with 0.6 (± 1.7). MUC5AC was not found in MuEp, ACC, EpMy, and SeC. The most likely explanation is that MUC5AC is, compared to MUC1 and MUC16, a type 2 secreted Mucin. MUC5AC seems not to qualify as a molecular target for salivary gland cancer therapy. As for MUC16, a significant association between the MUC5AC score and the female gender was found, most likely due to the gender distribution of MuEp and the MUC5AC combined score being the highest among MuEp compared to the other entities.

To date, in vitro data on therapeutic targets against MUC1 in SaDu cell-lines and xenograft models, MUC1 gene expression levels, and mechanisms for overexpression of MUC1 in SaDu are lacking. Therefore, the results of this study warrant further research regarding the abovementioned therapeutic targets against MUC1 in cell lines and animal models. Further, the limitation of retrospective collection of histopathological and clinical data must be considered when interpreting the results of this study.

Overall, the present study displays the extent and intensity of MUC1, MUC16, and MUC5AC among different entities of SGC. The results show that MUC1 is intensely expressed in salivary duct carcinoma, which is known for its aggressive growth and low survival rates while MUC16 shows the highest intensity in mucoepidermoid carcinoma.

## Data Availability

The datasets generated and analysed during the current study are available from the corresponding author on reasonable request.
